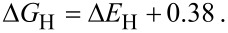# Improving the catalytic activity for hydrogen evolution of monolayered SnSe_2(1−_*_x_*_)_S_2_*_x_* by mechanical strain

**DOI:** 10.3762/bjnano.9.173

**Published:** 2018-06-18

**Authors:** Sha Dong, Zhiguo Wang

**Affiliations:** 1 School of Electronics Science and Engineering, Center for Public Security Technology Research, University of Electronic Science and Technology of China, Chengdu, 610054, P.R. China

**Keywords:** density functional theory (DFT), electronic properties, hydrogen evolution reaction, mechanical strain, SnSe_2(1−_*_x_*_)_S_2_*_x_* monolayer

## Abstract

Exploring efficient electrocatalysts for hydrogen production with non-noble metals and earth-abundant elements is a promising pathway for achieving practical electrochemical water splitting. In this work, the electronic properties and catalytic activity of monolayer SnSe_2(1−_*_x_*_)_S_2_*_x_* (*x* = 0–1) under compressive and tensile strain were investigated using density functional theory (DFT) computations. The results showed SnSe_2(1−_*_x_*_)_S_2_*_x_* alloys with continuously changing bandgaps from 0.8 eV for SnSe_2_ to 1.59 eV for SnS_2_. The band structure of a SnSe_2(1−_*_x_*_)_S_2_*_x_* monolayer can be further tuned by applied compressive and tensile strain. Moreover, tensile strain provides a direct approach to improve the catalytic activity for the hydrogen evolution reaction (HER) on the basal plane of the SnSe_2(1−_*_x_*_)_S_2_*_x_* monolayer. SnSeS and SnSe_0.5_S_1.5_ monolayers showed the best catalytic activity for HER at a tensile strain of 10%. This work provides a design for improved catalytic activity of the SnSe_2(1-_*_x_*_)_S_2_*_x_* monolayer.

## Introduction

Hydrogen is a clean energy source with outstanding properties such as high specific energy per mass, easy storage and transportation, and ability to reduce harmful emissions [1–2]. Hydrogen is not naturally available as a ready-to-use substance; however, hydrogen can be produced anywhere across the planet through many approaches. Among the approaches used in the mass production of hydrogen, water electrolysis is a clean and “green” approach [3–7]. Efficient electrocatalysts for the hydrogen evolution reaction (HER) with high conversion efficiency are essential for the continuous generation of hydrogen. The platinum (Pt) group materials are regarded as the best electrocatalysts for HER; however, the high cost and limited resources of these types of catalyst restrict their usage in the mass production of hydrogen [8–10]. Therefore, exploring non-noble and earth-abundant elements as catalysts for hydrogen production is one of the most promising pathways for the mass production of hydrogen.

Two-dimensional (2D) atomic layer thin materials, such as monolayer transition-metal dichalcogenides (TMDs) [11–19], have demonstrated many fascinating properties, including the substitution of Pt as an electrocatalyst for HER. 2D MoS_2_ has been widely studied. Recently, tin dichalcogenides SnX_2_ (X = S, Se) have also received considerable attention in a variety of fields because of their low cost, use of earth-abundant resources and environmental friendliness [18,20–23]. Monolayer SnX_2_ has a X–Sn–X sandwich-like structure, which can be easily synthesized by using traditional mechanical exfoliation techniques [24–25] because of the weak van der Waals (vdW) interactions between layers. These mono- and few-layer SnX_2_ (X = S, Se) compounds are expected to be widely used in the fields of water splitting [26], high-speed photodetection [27], electronics [28], and catalysis [29], as well as in the fabrication of solar cells and film electrodes [30].

A good electro-catalyst for HER should have sufficient active sites for catalysis. Furthermore, because electrons participate in the HER process, an ideal catalyst for HER should have good electronic conductivity. Tuning the band structure of the catalyst is important for improving the HER efficiency. It was reported that the band structure and carrier mobility of monolayer MX_2_ can be tuned by substitution of M with M' atoms or X with X' atoms to form monolayer M*_x_*M'_(1−_*_x_*_)_X_2_ or MX_2_*_x_*X'_2(1−_*_x_*_)_ alloys [31–37]. For example, Komsa et al. [34] have investigated the electronic properties of monolayer MoS_2_*_x_*Se_2(1−_*_x_*_)_ and found that the bandgaps can be continuously tuned with the variation of Se composition. Liu et al. [38] have studied Mo_1−_*_x_*W*_x_*S_2_ and observed variations of the direct bandgap between 1.85 and 1.99 eV by varying *x* from 0 to 1. Other 2D-TMDs alloy nanosheets, such as Mo_1−_*_x_*W*_x_*Se_2_ [39] and WS_2(1−_*_x_*_)_Se_2_*_x_* [40–41], have also shown tuneable bandgaps and different electrical properties by varying the value of *x*. Monolayer SnS_2_ and SnSe_2_ have indirect bandgaps of 2.1 and 1.1 eV, respectively. Theoretically, alloying SnS_2_ and SnSe_2_ may yield tuneable bandgaps [36]. Wang et al. [35] have studied the 2D SnSe_2(1−_*_x_*_)_S_2_*_x_* alloys that were alloyed using chemical vapour transport (CVT) reactions.

Apart from alloying, strain can also be used to tune the electronic properties of nanomaterials. Yue et al. [42] investigated the electronic properties of monolayer MoS_2_ under elastic strain and found that the direct-to-indirect transition of MoS_2_ occurs at a strain of 0.01, and the semiconductor-to-metal transition occurs at a strain of 0.10. Huang et al. [22] reported that both compressive (−11%) and tensile (14%) strain can trigger the semiconductor–metal transition in the SnSe_2_ monolayer. Furthermore, Scalise et al. [15] showed that the electronic structure of the MoS_2_ monolayer can be reversibly tuned from direct to indirect by applying strain (ca. 2%).

Much research effort has been devoted in recent decades to the development of inexpensive catalysts for the electrochemical HER. Some of the recent studies have focused on monolayer SnX_2_ (X = S, Se). Liu et al. [43] investigated SnS_2_ nanosheets regarding their electrochemical behaviour and electrocatalytic properties for HER by examining trace amounts of Pt nanoparticles interacting with defect-rich SnS_2_; the results demonstrated that SnS_2_ may offer new perspectives regarding a utilization in HER. The catalytic activity for HER shows great dependence on the electronic structure of the catalyst. As alloying and strain can be used to tune the electronic properties of the catalyst, they will affect the catalytic behaviour. Several TMD alloy systems have been investigated as catalysts for HER, including Mo_1−_*_x_*W*_x_*Se_2_ nanoflowers [44], WS_2(1−_*_x_*_)_Se_2_*_x_* nanotubes [45] and MoS_2(1−_*_x_*_)_Se_2_*_x_* nanobelts [46]. It was reported that alloying provides an opportunity to tune the Gibbs free energy (Δ*G*_H_) for hydrogen adsorbed on the monolayer alloys and can be used to enhance HER performance. The HER catalytic properties of the catalyst can also be tuned through strain [42,47–52]. Gao et al. [53] demonstrated that a tensile strain is able to strengthen the hydrogen binding on graphitic carbon nitride (g-C_3_N_4_), whereas compressive strain had the opposite effect. Yan et al. [49] showed that large elastic strains influence the catalytic activity of WC for HER.

Very recently, 2D SnSe_2(1−x)_S_2x_ alloys have been synthesized experimentally [35]. To our knowledge, theoretical studies related to the system of 2D SnSe_2(1−_*_x_*_)_S_2_*_x_* alloys as catalysts for HER have been reported rarely. Tuning the electronic properties and catalytic behaviour of SnSe_2(1−_*_x_*_)_S_2_*_x_* monolayers for HER by strain engineering is required for their application in the energy conversion field. In this work, the electronic properties and catalytic behaviour for HER of SnSe_2(1−_*_x_*_)_S_2_*_x_* (*x* = 0, 0.125, 0.25, 0.375, 0.5, 0.625, 0.750, 0.875 and 1.0) monolayers were investigated by density functional theory (DFT). It was shown that band gap and catalytic activity of these alloys can be continuously tuned by strain engineering.

## Results and Discussion

**Figure 1:**
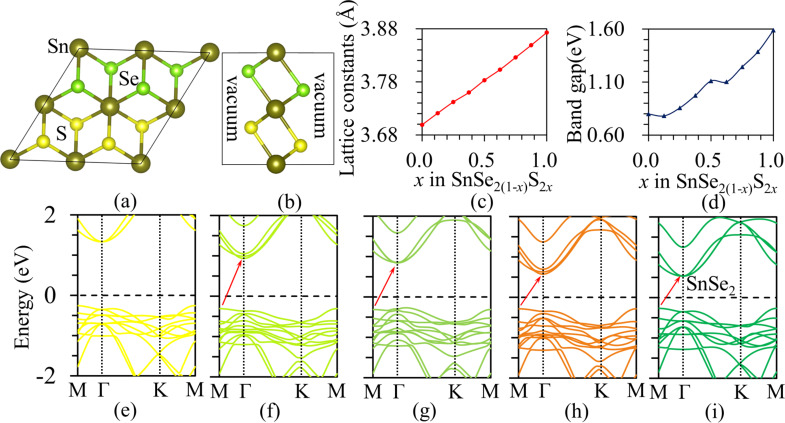
(a) Top view and (b) side view of monolayer SnSeS. (c) Variation of the lattice constant as a function of Se content in monolayer SnSe_2(1−_*_x_*_)_S_2_*_x_*. (d) Band gaps as a function of Se content in monolayer SnSe_2(1−_*_x_*_)_S_2_*_x_*. (e–i) The band structures of monolayer SnSe_2(1−_*_x_*_)_S_2_*_x_* with *x* equal to (e) 1.0, (f) 0.75, (g) 0.50, (h) 0.25 and (i) 0.0. The arrows indicate the indirect band gap for a given system.

2D monolayer TMD is a three-atomic thickness structure with one transition-metal atom layer sandwiched by two chalcogen atom layers. A triangular prism or octahedron can be formed as the transition-metal atom is bonded with six chalcogen atoms, and the former and latter structures are often termed as 2H-phase and 1T-phase, respectively. The stable phase for monolayer SnS_2_ is the 1T-phase. Alloying is an efficient approach to tune the electronic properties of semiconductors. Because of the large difference of bandgaps between SnSe_2_ and SnS_2_, the SnSe_2(1−_*_x_*_)_S_2_*_x_* semiconductor offers wide and continuously fine-tuneable bandgaps when varying the alloy fractions. All the simulations were performed in a 2 × 2 supper cell, which includes 8 S atoms. As 0–8 S atoms are substituted by Se atoms, the monolayer SnSe_2(1−_*_x_*_)_S_2_*_x_* alloys with *x* = 0–1 are formed. For each given S content, all possible configurations of the substitution of S by Se atoms in monolayer SnS_2_ are tested; the configuration with the lowest energy was used to model the monolayer SnSe_2(1−_*_x_*_)_S_2_*_x_*, which is different from random structure used in [36]. The electronic properties and catalytic behaviour for HER of the SnSe_2(1−_*_x_*_)_S_2_*_x_* monolayer are all performed for the energy-stable configuration. The cross and side views of monolayer SnSeS are shown in Figure 1a and Figure 1b, respectively. Sn, S, and Se atoms are presented by brown, yellow, and green balls, respectively. For the monolayer SnSeS, S and Se atoms are preferably distributed orderly. As the size of the S atom is different from that of Se atom, the lattice constant of SnSe_2(1−_*_x_*_)_S_2_*_x_* monolayer changes with the Se content. Figure 1c shows the variation of the lattice constant as a function of the Se content as *x* increases from 0 to 1. The lattice constant decreases from 3.87 Å for SnSe_2_ to 3.70 Å for SnS_2_. The calculated lattice constants of 3.87 and 3.70 Å agree with the reported values of 3.89 Å for SnSe_2_ and 3.70 Å for SnS_2_ [35]. The lattice constant shows a linear variation with Se content, which obeys Vegard′s law [54] and agrees well with previous results [36].

SnS_2_ and SnSe_2_ monolayers are indirect-bandgap semiconductors, as highlighted in their band structures shown in Figure 1e and Figure 1i, respectively. The valence-band maximum (VBM) is located at the M-point, whereas the conduction-band minimum (CBM) is located at the Γ-point. The band gaps are 1.59 eV for the SnS_2_ monolayer and 0.80 eV for the SnSe_2_ monolayer, in agreement with the previous reported values of 1.60 eV and 0.81 eV, respectively [35]. The band structures of monolayer SnSe_2(1−_*_x_*_)_S_2_*_x_* with *x* equal to 0.75, 0.50 and 0.25 are shown in Figure 1f, Figure 1g and Figure 1h, respectively. The substitution of S with Se does not affect the indirect bandgap semiconducting characteristics; however, the band gap is tuned with changing the content of Se, as shown in Figure 1d with the indirect band gap decreasing with increasing Se content. These results agree with the reported by Huang et al. [36] with random SnSe_2(1−_*_x_*_)_S_2_*_x_* alloys. The bandgap evolution of monolayer SnSe_2(1−_*_x_*_)_S_2_*_x_* shows a band-bowing effect [36].

**Figure 2:**
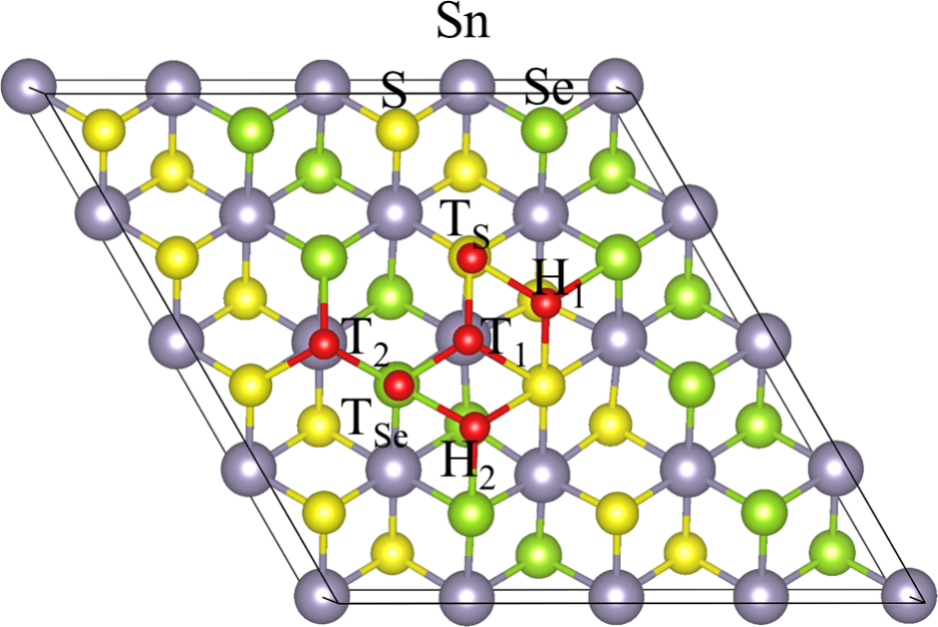
Atom configuration of possible adsorption sites for hydrogen on the SnSeS monolayer.

There are three possible adsorption sites for hydrogen on pristine SnS_2_ and SnSe_2_ monolayers, i.e., the top of the Sn site, the centre of a hexagonal site, and the top of S/Se atoms. Because of the symmetry breaking in SnSe_2(1−_*_x_*_)_S_2_*_x_* monolayers, the number of possible adsorption sites is increased. For example, there are six adsorption sites for hydrogen on the SnSeS monolayer, as shown in Figure 2, i.e., the top of S (T_S_) and Se (T_Se_) atoms, two tops of the Sn sites (T1 and T2), and two centres of the hexagonal sites (H1 and H2). The hydrogen is bounded to two S atoms and one Se atom at the T1 and H1 sites, whereas it is bounded to one S atom and two Se atoms at the T2 and H2 sites. After full relaxation, the T and H sites are not energetically stable adsorption sites for hydrogen, which will be relaxed to near the S or Se atoms. Thus, we considered the sites on top of the S and Se atoms as adsorption sites for hydrogen in the following part of this paper.

The Gibbs free energies of hydrogen adsorption on the basal plane of the monolayer of SnS_2_ and SnSe_2_ are 1.08 and 0.96 eV, respectively. Although the Gibbs free energies of hydrogen adsorption at on top of Se and S atoms decrease with increasing the Se content in the SnSe_2(1−_*_x_*_)_S_2_*_x_* monolayer, the values of Δ*G*_H_ are 0.99 and 0.61 eV on top of Se and S atoms, respectively, in the SnSe_1.75_S_0.25_ monolayer. The large positive values of Δ*G*_H_ indicate that hydrogen has a weak interaction with the substrate and is not easily adsorbed on the catalyst. Thus, the basal plane of SnSe_2(1−_*_x_*_)_S_2_*_x_* monolayer remains chemically inert for HER.

**Figure 3:**
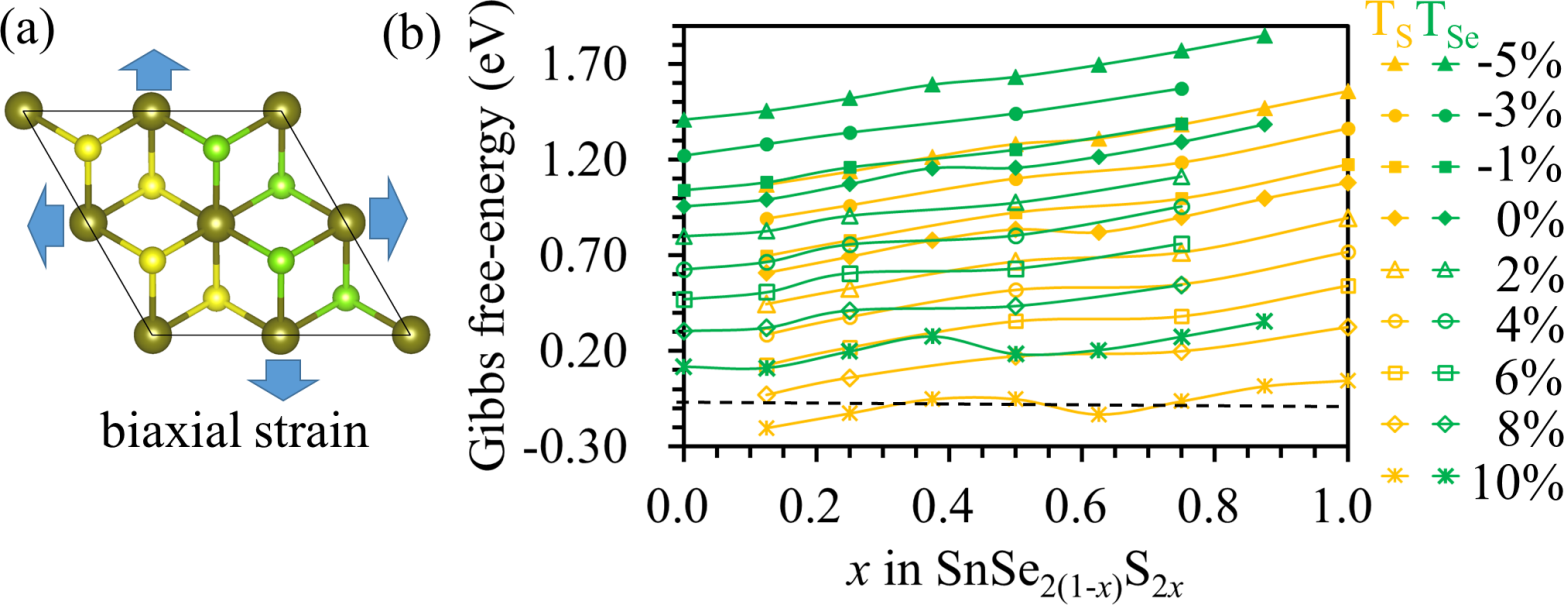
(a) A schematic diagram showing strain applied to SnSe_2(1−_*_x_*_)_S_2_*_x_* monolayer. (b) Evolution of Δ*G*_H_ for the SnSe_2(1−_*_x_*_)_S_2_*_x_* monolayer with mechanical strain. The black dashed line indicates a Gibbs free energy of zero.

As shown in Figure 3a, biaxial strains ranging from −5% to 10% were applied in the SnSe_2(1−_*_x_*_)_S_2_*_x_* monolayer to modify the catalytic performance. Strained SnSe_2(1−_*_x_*_)_S_2_*_x_* monolayer are obtained by varying the lattice value with strain ε (ε = −0.05, −0.03, −0.01, 0.02, 0.03, 0.05, 0.06 and 1.00), i.e., the lattice constant is *a* = *a*_0_(1 + ε), where *a*_0_ is the lattice constant without strain. The value of Δ*G*_H_ for hydrogen absorbed on SnSe_2(1−_*_x_*_)_S_2_*_x_* monolayers increases with compressive strains. The values of Δ*G*_H_ were calculated to be 0.84 and 1.16 eV in the SnSeS monolayer for hydrogen adsorbed on top of S and Se atoms, respectively, and these values increased to 1.28 and 1.63 eV, respectively, under a compressive strain of −5%. Thus, compressive strain is not helpful for improving the catalytic performance of the SnSe_2(1−_*_x_*_)_S_2_*_x_* monolayers. With tensile strain applied, the value of Δ*G*_H_ decreases with increasing the tensile strain. The values of Δ*G*_H_ decrease from 0.84 to −0.05 eV and from 1.16 to 0.18 eV for hydrogen adsorbed on top of the S atom and the Se atom, respectively, in the SnSeS monolayer as the tensile strain increases from 0% to 10%. For the SnSe_2(1−_*_x_*_)_S_2_*_x_* monolayer with *x* in the range between 0.25 and 1.00, the values of Δ*G*_H_ for hydrogen adsorbed the top of S are close to zero at a tensile strain of 10%; thus, strain can be used to improve the catalytic activity for HER.

**Figure 4:**
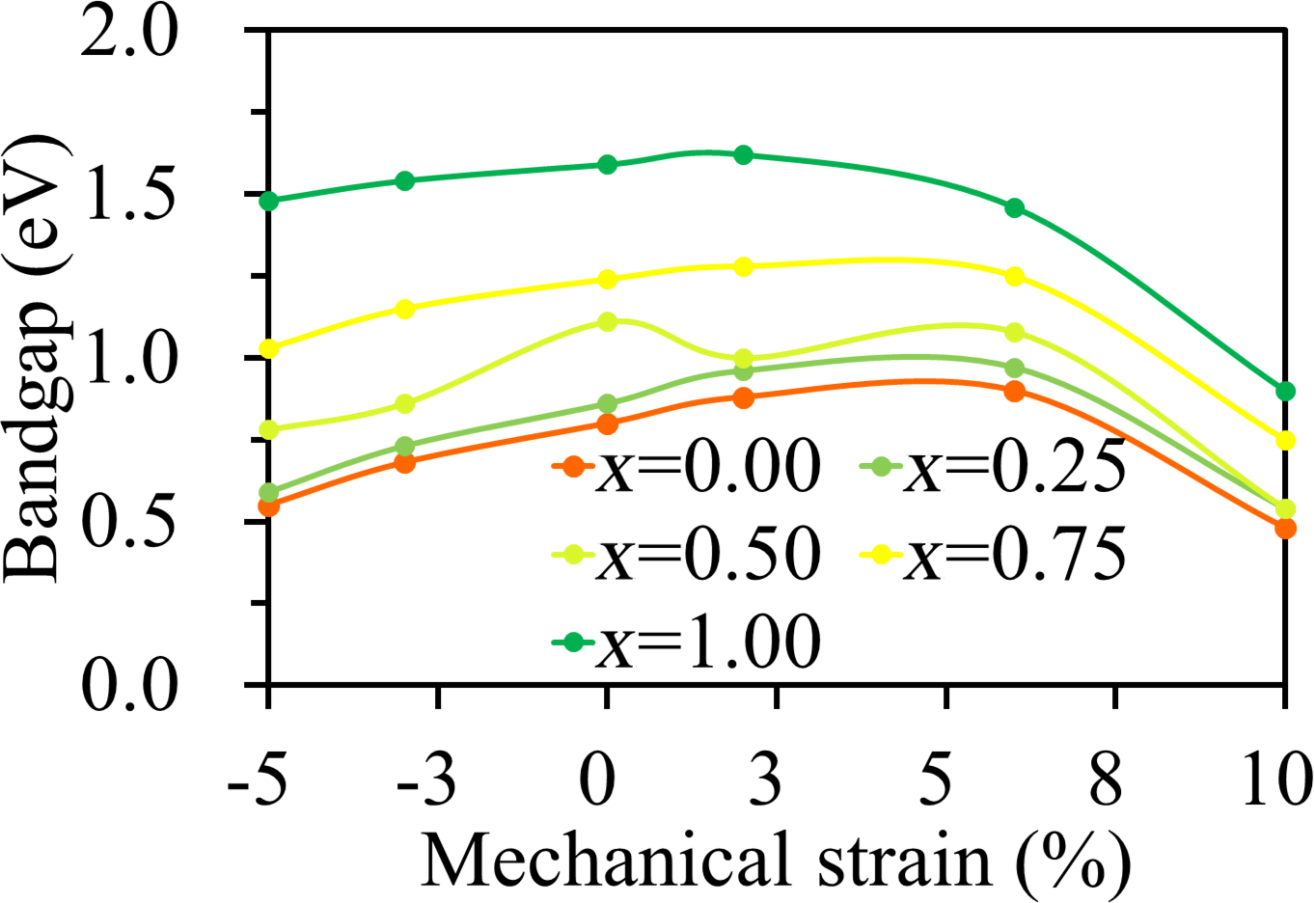
Band gaps of the SnSe_2(1−_*_x_*_)_S_2_*_x_* monolayers as a function of mechanical strain.

As shown in Figure 4, also the band gaps show great dependence on the applied strain. The band gap decreases from 1.59 to 1.48 eV for the SnS_2_ monolayer as the compressive strain increases from 0% to −5%, whereas it decreases from 1.59 to 0.90 eV as the tensile strain increases from 0% to 10%. The results indicates that the strain can be used to tune the band gaps of the SnSe_2(1−_*_x_*_)_S_2_*_x_* monolayers.

**Figure 5:**
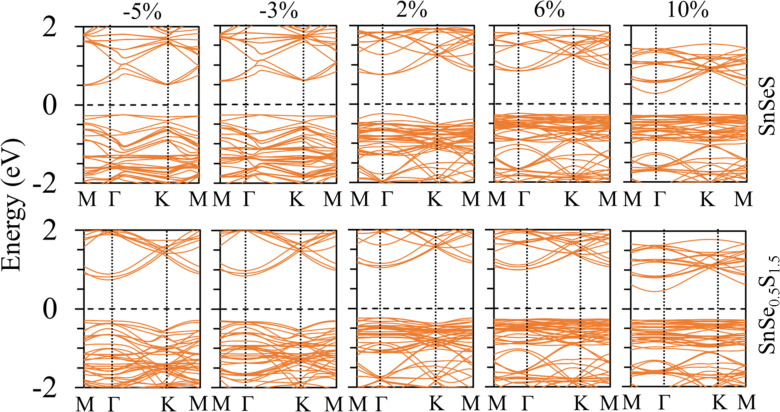
Band structures for SnSeS and SnSe_0.5_S_1.5_ monolayers with strain of −5%, −3%, 2%, 6% and 10%.

These effects may create new opportunities in some specific applications, such as bandgap engineering and device structures. It was reported that the catalytic activity for HER of the monolayer 1T'-MX_2_ (M = Mo, W; X = S, Se, Te) can be enhanced by applying tensile strain [55–56]. For 1T'-MoS_2_, it was found that the strong hybridization between Mo d-orbitals and S p-orbitals increases upon application of tensile strain because the valence band and conduction band move upward and downward in energy, respectively. Previous reports showed that H adsorption depends on the density of states near the Fermi energy level [57–58], with the adsorption being enhanced as the d-band centre moves closer to the Fermi level. The number of electronic states near the Fermi energy increased with the increasing of tensile mechanical strain, thereby boosting the supply of electrons to the adsorption sites and thus improving the catalytic activity [59]. Figure 5 shows the band structures for SnSeS and SnSe_0.5_S_1.5_ monolayers with strains of −5%, −3%, 2%, 6% and 10%. As seen from Figure 5, there are fewer valence states near the Fermi energy when compressive strain is applied, and the number of electronic states increases with increasing tensile strain. A higher density of electronic states near the valence-band maximum appears with tensile strain. Moreover, tensile strain caused the valence band to shift upward and the conduction band to shift downward in energy. The results can enhance the interaction between hydrogen and the catalyst. Therefore, the hydrogen-adsorption free energies were decreased from 0.84 to −0.05 eV and from 0.90 to −0.06 eV for H atoms adsorbed on SnSeS and SnSe_0.5_S_1.5_ at a tensile strain of 10%, respectively. Therefore, applying a suitable tensile strain can efficiently improve the HER efficiency of SnSe_2(1−_*_x_*_)_S_2_*_x_* monolayers. The tensile strain can be realized by mechanically bending the monolayer SnSe_2(1−_*_x_*_)_S_2_*_x_* [56].

## Conclusion

In conclusion, the electronic properties and catalytic behaviour for HER of SnSe_2(1−_*_x_*_)_S_2_*_x_* monolayers were investigated using DFT calculations. The band gap of the SnSe_2(1−_*_x_*_)_S_2_*_x_* monolayer can be continuously tuned from 0.8 eV for SnSe_2_ to 1.59 eV for SnS_2_. The band gap of a SnSe_2(1−_*_x_*_)_S_2_*_x_* monolayer can be further tuned by applying mechanical strain. Although the basal plane of SnSe_2(1−_*_x_*_)_S_2_*_x_* monolayer is inert for HER, the mechanical tensile strain provides a direct means to improve the catalytic activity for hydrogen evolution reaction of SnSe_2(1−_*_x_*_)_S_2_*_x_* monolayer. SnSeS and SnSe_0.5_S_1.5_ monolayers show the best catalytic activity for HER at a tensile strain of 10%. This work provides a method of improvement of the catalytic activity of SnSe_2(1−_*_x_*_)_S_2_*_x_* monolayers.

## Simulation Details

All calculations were performed based on spin-polarized DFT as implemented in the Vienna ab initio simulation package (VASP) [60–61] within the framework of the projector augmented wave method [62]. Electron exchange–correlation was described with the generalized gradient approximation (GGA) within the Perdew–Burke–Ernzerhof (PBE) functional [63]. An energy cutoff of 520 eV was used for the plane-wave basis sets to converge the relevant quantities. The atomic positions and geometric structures were freely relaxed using the conjugate gradient approximation (CG) until the residual force on each atom is less than 0.02 eV·Å^−1^. A vacuum of 20 Å perpendicular to the monolayer was used to avoid the periodic image interactions.

The HER can be described by Equation 1 under the conditions pH = 0 and *p*(H_2_) = 1bar [64]:

[1]
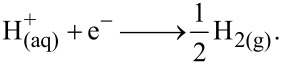


The first step of HER is that the H atom is bound to the active site of the catalyst; this step is the rate-determining step [65]. The Gibbs free energy of hydrogen adsorption (Δ*G*_H_) of this step is a key quantity to describe the HER activity of the catalyst; a value of Δ*G*_H_ for a good catalyst should be close to zero [66–68]. Δ*G*_H_ can be calculated by using Equation 2,

[2]
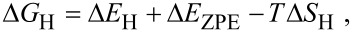


where Δ*E*_ZPE_ is the zero-point energy difference between the adsorbed state and the gas phase of hydrogen; because this quantity has a small contribution to Δ*G*_H_, it was neglected in this work. *T* is the temperature. Δ*S*_H_ is the entropy contribution to Δ*G*_H_, which can be approximated as: Δ*S*_H_ = 1/2*S*_H2_, where *S*_H2_ is the entropy of H_2_ in the gas phase under standard conditions (300 K, 1 bar) [69]. The approximate value of Δ*E*_ZPE_ − *T*Δ*S*_H_ is 0.38 eV [70–71]. Δ*E*_H_ is the hydrogen chemisorption energy and was calculated using Equation 3,

[3]
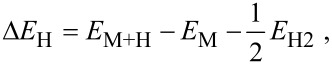


where *E*_M+H_, *E*_M_, and *E*_H2_ are the total energy of the catalyst with an adsorbed hydrogen atom, the total energy of the catalyst without adsorption of hydrogen, and the total energy of a molecule of hydrogen, respectively. Using the above values, the Gibbs free energy can be calculated using Equation 4,

[4]